# MiR-200c inhibits bladder cancer progression by targeting lactate dehydrogenase A

**DOI:** 10.18632/oncotarget.18801

**Published:** 2017-06-28

**Authors:** Daozhang Yuan, Shunsheng Zheng, Liyan Wang, Jing Li, Jianan Yang, Bin Wang, Xiong Chen, Xiaobo Zhang

**Affiliations:** ^1^ Department of Urology, Cancer Center of Guangzhou Medical University, Guangzhou, People's Republic of China; ^2^ Department of Geriatric Medicine, Xiangya Hospital, Central South University, Changsha, People's Republic of China

**Keywords:** microRNA, miR-200c, LDHA, bladder cancer, proliferation

## Abstract

Lactate dehydrogenase A (LDHA) is overexpressed in various cancers. We investigated LDHA expression and function in bladder cancer. We demonstrate that LDHA is up-regulated in bladder cancer cells and promotes proliferation, invasion, and glycolysis. Additionally, we found that microRNA (miR)-200c directly targets LDHA in bladder cancer cells. Ectopic expression of miR-200c inhibited LDHA-induced glycolysis, cell proliferation, and invasion. Thus, targeting LDHA through miR-200c is a potential therapeutic strategy in bladder cancer.

## INTRODUCTION

Bladder cancer is the leading genitourinary malignancy [1]. There were an estimated 76,960 new cases and 16,390 bladder cancer-related deaths in the United States in 2016 [2]. Although approximately 75% of new cases are noninvasive subtypes, the recurrence rate is high following local treatment [3].

MicroRNAs (miRNAs) are small, endogenous, single-stranded RNAs with various functions [4]. MiRNAs bind to highly conserved, complementary sequences in the 3′-untranslated regions (3′-UTRs) of target mRNAs and inhibit protein translation. MiRNAs can also regulate gene expression by inducing degradation of target mRNAs [5]. MiRNAs have important roles in tumor development and progression, and are correlated with prognosis in various cancers [6, 7]. MiR-200c regulates epithelial-to-mesenchymal transition (EMT) and chemosensitivity [8, 9]. Dysregulation of miR-200c has been observed in several cancers including gastric [10], breast [11], and renal cell carcinoma [12].

The Warburg effect is a hallmark of solid tumors. It is characterized by continuous conversion of glucose into lactate under adequate oxygen conditions (aerobic glycolysis) [13, 14] and is correlated with cancer cell proliferation and metastasis [15]. Lactate dehydrogenase (LDH) catalyzes the conversion of pyruvate to lactate. LDH is comprised of four subunits. The most common subunits are LDHA (muscle-type, M subunit) and LDHB (heart-type, H subunit) [16]. LDHA has a critical role in aerobic glycolysis. Elevated LDHA expression was observed in several cancers and was associated with worse overall survival [17, 18]. LDHA expression is regulated by several oncogenes and deacetylases including MYC and HIF-1α. Therefore, it is a potential therapeutic target [19].

We investigated LDHA expression and function in bladder cancer. Additionally, we analyzed the association between miR-200c and LDHA expression. Our data indicate miR-200c is a tumor suppressor that directly targets LDHA.

## RESULTS

### LDHA is up-regulated and promotes cell proliferation and invasion in bladder cancer

**Figure 1:**
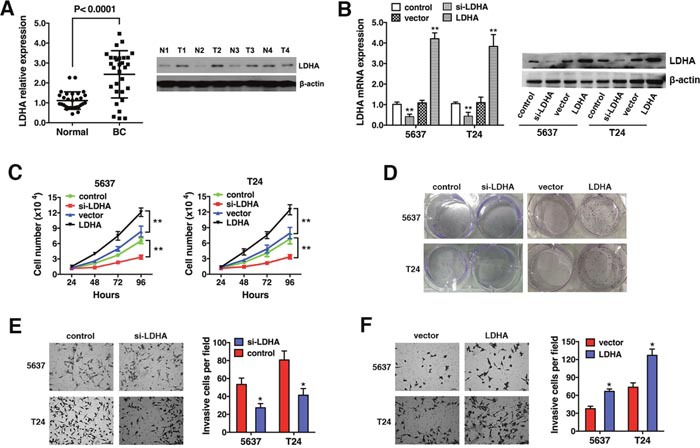
LDHA is up-regulated and promotes cell proliferation and invasion in bladder cancer **(A)** LDHA expression in 30 bladder cancer (BC) and 30 matched normal bladder (Normal) samples was assessed by qRT-PCR (left) and Western blotting (right). β-actin was used as an endogenous control. **(B)** T24 and 5637 cells were transfected with an LDHA expression vector or si-LDHA. **(C)** Analysis of cell proliferation 24, 48, 72, and 96 hours after transfection. **(D)** *In vitro* colony formation assays. **(E)** Transwell invasion assays of cells transfected with si-LDHA or control. **(F)** Transwell assays of cells transfected with either the LDHA expression vector or control. Data are presented as the mean ± s.e.m. * *P* < 0.05, ** *P* < 0.01.

LDHA expression was evaluated in 30 bladder cancer (BC) and 30 matched normal bladder (Normal) samples by qRT-PCR. We found that LDHA was up-regulated in bladder cancer tissue (Figure 1A, left). Up-regulation of LDHA was confirmed by Western blot analysis of four bladder cancer and four matched normal bladder samples (Figure 1A, right). We transfected T24 and 5637 cells with either an LDHA expression vector or si-LDHA. LDHA levels were confirmed by qRT-PCR and Western blotting (Figure 1B). Cell proliferation and colony formation assays were performed to assess the effect on bladder cancer cell growth. Overexpression of LDHA increased, while down-regulation decreased, cell proliferation (Figure 1C and 1D). Down-regulation of LDHA suppressed cell invasion (Figure 1E, while overexpression enhanced the invasive capacity of bladder cancer cells (Figure 1F). Thus, LDHA is up-regulated in bladder cancer and it promotes cell proliferation and invasion.

### LDHA promotes glycolysis in bladder cancer

**Figure 2:**
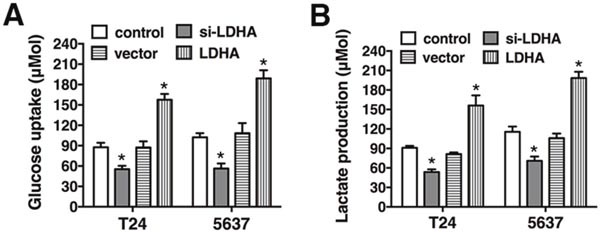
LDHA promotes glycolysis in bladder cancer **(A)** Glucose uptake in T24 and 5637 cells transfected with the LDHA expression vector or si-LDHA. **(B)** Lactate production after transfection. Data are presented as the mean ± s.e.m. * *P* < 0.05.

To assess the metabolic effects of LDHA in bladder cancer cells, T24 and 5637 cells were transfected with either the LDHA expression vector or si-LDHA. Overexpression of LDHA resulted in increased glucose uptake and lactate production, while decreased glucose uptake and lactate production were observed in cells transfected with si-LDHA. These data indicate LDHA promotes aerobic glycolysis in bladder cancer (Figure 2).

### LDHA is a direct target of miR-200c and is negatively correlated with miR-200c in bladder cancer

**Figure 3:**
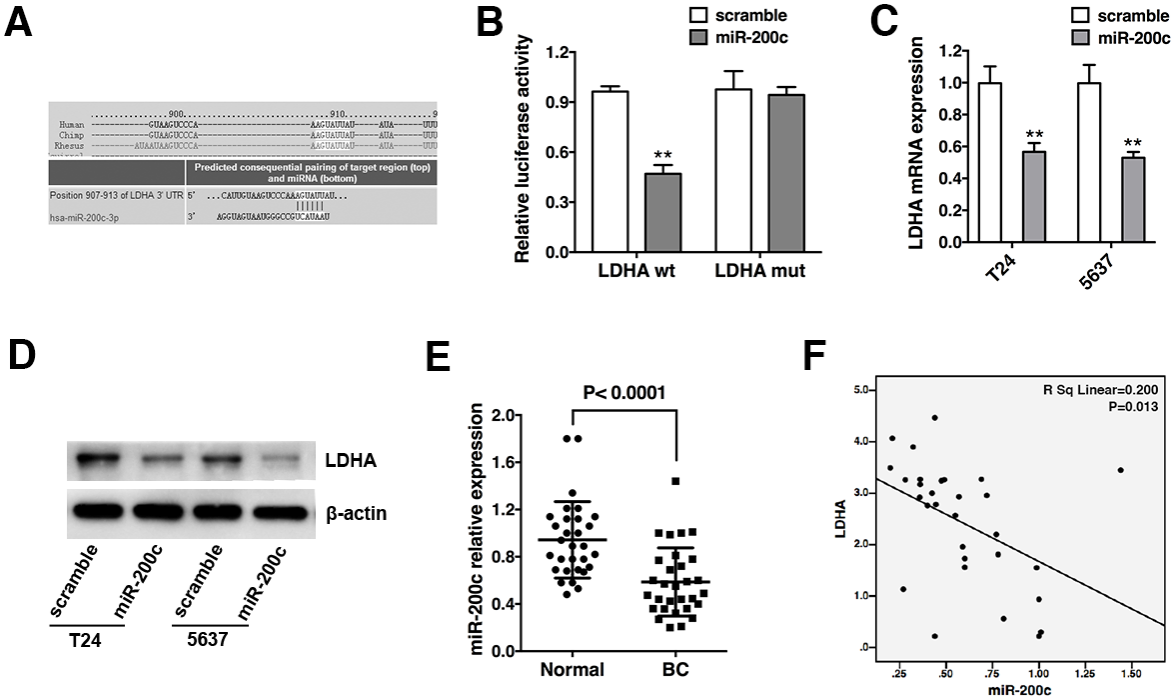
LDHA is a direct target of miR-200c and is negatively correlated with miR-200c in bladder cancer **(A)** The predicted binding site of miR-200c in the LDHA mRNA. **(B)** Co-transfection of T24 cells with miR-200c mimics or scramble and a luciferase reporter containing the full-length or mutant LDHA 3’-UTR (LDHA-wt or LDHA-mut). Luciferase activity was measured 48 hours after transfection. **(C)** Analysis of LDHA mRNA levels in T24 and 5637 cells transfected with miR-200c mimics or scramble by qRT-PCR. β-actin mRNA was used as an endogenous control for normalization. **(D)** T24 and 5637 cells were transfected as described and the LDHA protein level measured by Western blotting. β-actin was used as an endogenous control for normalization. **(E)** Analysis of miR-200c expression in 30 bladder cancer (BC) and 30 matched normal (Normal) bladder samples by qRT-PCR. U6 snRNA was used as an endogenous control. **(F)** Quantitative RT-PCR indicates LDHA mRNA levels are inversely correlated with miR-200c. Data are presented as the mean ± s.e.m. ** *P* < 0.01.

TargetScan, PicTar, and miRanda were used to identify miRNAs that directly bind to the 3’-UTR of LDHA mRNA. A binding site for miR-200c was identified (Figure 3A). We performed luciferase reporter assays in T24 bladder cancer cells to determine whether LDHA was a direct target of miR-200c. The full-length LDHA 3’-UTR was sub-cloned into a luciferase reporter vector and co-transfected with miR-200c mimics or scramble control. Co-transfection of T24 cells with the wild-type 3’-UTR of LDHA and miR-200c mimics resulted in an approximately 50% reduction in luciferase activity (Figure 3B). Additionally, mutation of the putative miR-200c sites in the 3’-UTR of LDHA abrogated the luciferase response to miR-200c (Figure 3B). These results were confirmed by qRT-PCR and Western blotting. We also observed a reduction in LDHA mRNA and protein in cells transfected with miR-200c mimics (Figure 3C and 3D). MiR-200c expression was analyzed in 30 bladder cancer and matched normal bladder tissue samples. MiR-200c was down-regulated in bladder cancer tissue (Figure 3E), and a negative correlation was observed between LDHA expression and miR-200c (Figure 3F). Therefore, LDHA is a direct target of miR-200c in bladder cancer.

### MiR-200c inhibits LDHA-induced glycolysis, cell proliferation, and invasion

**Figure 4:**
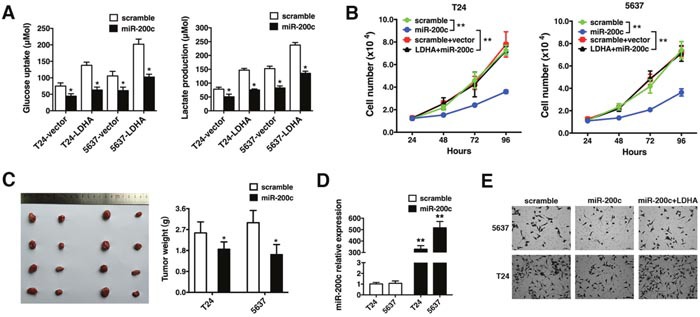
MiR-200c inhibits LDHA-induced glycolysis, cell proliferation, and invasion **(A)** Glucose uptake (left) and lactate production (right) 48 hours after transfection. **(B)** T24 and 5637 cells were transfected with scramble, miR-200c mimics, vector + scramble, or the LDHA expression vector + miR-200c mimics and the number of cells quantified 24, 48, 72, and 96 hours after transfection. **(C)** T24 or 5637 cells were injected subcutaneously and the mice randomly assigned to scramble or miR-200c mimic groups (four mice per group). Mice were sacrificed after 28 days and necropsies performed. Xenograft tumors were collected and weighed. **(D)** Detection of miR-200c in xenograft tumors by qRT-PCR. **(E)** Transwell assays of T24 and 5637 cells transfected with scramble, miR-200c mimics, or an LDHA expression vector + miR-200c mimics. Data are presented as the mean ± s.e.m. * *P* < 0.05, ** *P* < 0.01.

We investigated whether miR-200c inhibited glycolysis, cell proliferation, or invasion in bladder cancer cells by targeting LDHA. T24 and 5637 cells were transfected with either a control or LDHA expression vector followed by scrambled control or miR-200c mimics. The LDHA-induced increase in glycolysis was repressed by miR-200c, leading to a decrease in glucose uptake and lactate production (Figure 4A). Additionally, miR-200c overexpression inhibited LDHA-induced cell proliferation (Figure 4B).

We performed xenograft experiments to confirm the effects of miR-200c on tumor growth. T24 or 5637 cells were injected subcutaneously. Mice were randomly assigned to scramble or miR-200c mimic groups (four mice per group). The mice were sacrificed after 28 days and tumors weighed. Ectopic expression of miR-200c decreased tumor size and weight (Figure 4C). The presence of miR-200c in xenograft tumors was confirmed by qRT-PCR (Figure 4D). Transwell assays revealed that miR-200c abrogated LDHA-induced invasion (Figure 4E). Thus, miR-200c suppresses LDHA-induced glycolysis, cell proliferation, and invasion in bladder cancer.

## DISCUSSION

We found that LDHA is up-regulated in bladder cancer and that it acts as an oncogene. We also demonstrated that miR-200c targets LDHA and inhibits cell proliferation, invasion, and glycolysis in bladder cancer cells. Cancer cells convert glucose to lactate under aerobic conditions to support rapid proliferation [13]. Increased glucose uptake and lactate production have been observed in response to LDHA overexpression. Therefore, suppression of LDHA could inhibit tumor progression [20, 21].

MiRNAs can regulate gene expression by binding to the 3’-UTRs of target mRNAs [5]. Previous studies have demonstrated that miRNAs influence cell proliferation, differentiation, and metabolism, and abnormal miRNA expression has been detected in several cancers [10–12]. The miR-200 family includes miR-200a, miR-200c, miR-200c, miR-141, and miR-429, which share the same seed sequence and homologous targets. Low miR-200c expression was observed in invasive bladder cancer and predicts poor prognosis [22, 23]. MiR-200c was shown to inhibit invasion, migration, and proliferation of bladder cancer cells through down-regulation of BMI-1 and E2F3 [24]. Additionally, miR-200 regulates EMT in bladder cancer cells through decreasing ZEB1 expression [25, 26]. Treatment of T24 bladder cancer cells with miR-200c led to decreased proliferation and migration, cell cycle alterations, and enhanced doxorubicin sensitivity [27]. We found that miR-200c was down-regulated in bladder cancer and that it suppressed glycolysis, proliferation, and invasion.

We found that miR-200c expression was inversely correlated with LDHA expression in bladder cancer. MiR-200c directly targets LDHA to suppress cell glycolysis, proliferation, and invasion in bladder cancer. Overexpression of miR-200c inhibits cell proliferation, invasion, and glycolysis by targeting LDHA. Inhibition of LDHA may be an effective therapeutic strategy in bladder cancer.

## MATERIALS AND METHODS

### Patient samples

Tissue samples were sectioned and stored in RNAlater (Ambion, Austin, TX, USA) for quantitative real-time PCR (qRT-PCR) analysis. Patients were diagnosed with bladder cancer between March 2009 and March 2014 at the Accessory Cancer Center of Guangzhou Medical University. This study was approved by the Ethics Committee of the Accessory Cancer Center of Guangzhou Medical University Health Authority. Informed consent was obtained from all patients. The study was performed in accordance with the Declaration of Helsinki.

### Cell lines and culture

The T24 and 5637 human bladder cancer cell lines were obtained from the Chinese Academy of Medical Science (Beijing, China) and cultured in RPMI-1640 medium supplemented with 10% fetal calf serum and penicillin-streptomycin. All cells were maintained at 37°C in 5% CO_2_ and 95% air.

### Quantitative RT-PCR

Total RNA was extracted with the TRIzol reagent (Invitrogen, Carlsbad, CA, USA). LDHA cDNA was synthesized from 1 μg of total RNA using the Reverse Transcription System (Promega, Madison, WI, USA). β-actin was amplified in parallel as an internal control. For miR-200c, reverse transcription and qRT-PCR were performed using a qSYBR-green-containing PCR kit (GenePharma, Shanghai, China). U6 snRNA was used as an endogenous control. Gene expression was quantified by measuring cycle threshold (Ct) values and normalized to β-actin or U6 snRNA using the 2^−ΔΔCt^ method.

### Cell proliferation assays

T24 and 5637 cells were plated in six-well plates and transfected with an LDHA expression vector or si-LDHA. Cells were counted 24, 48, 72, and 96 hours after transfection using a Coulter Counter (Beckman Coulter, Fullerton, CA, USA). All experiments were performed in triplicate.

### Colony formation assays

T24 and 5637 cells were transfected with an LDHA expression vector or si-LDHA. Following transfection, cells were counted and seeded in six-well plates in triplicate (150 cells per well). Culture medium was replaced every 3 days. Colonies were only counted if they contained more than 50 cells. The number of colonies was analyzed 6 days after seeding.

### Transwell assays

Cells were seeded onto inserts coated with a basement membrane matrix in 24-well plates (EC matrix, Chemicon, Temecula, CA, USA). After 48 hours, the non-invaded cells and EC matrix were gently removed with a cotton swab. Invaded cells located on the lower side of the chamber were stained with crystal violet, counted, and imaged.

### Measurement of glucose consumption and lactate production

T23 and 5634 cells were transfected with either the LDHA expression vector or si-LDHA. Cell culture media was collected 48 hours after transfection. Glucose uptake and lactate production were measured using the Amplex® Red Glucose/Glucose Oxidase Assay Kit (Invitrogen) and the Lactate Assay Kit (Sigma, St. Louis, MO, USA), respectively. The results were normalized to total cellular protein.

### Luciferase assays

The full-length LDHA 3′-UTR (LDHA-wt, CAUUGUAAGUCCCAAAGUAUUAU, 5'→3') was amplified and cloned into the Sacl and Mlul sites of the pMIR-REPORT luciferase vector (Ambion). As a control, an LDHA mutant vector (LDHA-mut, CAUUGUAAGUCCCAACAAUCCAU, 5'→3'), was generated in which the first five nucleotides complementary to the miR-200c seed-region were mutated by site-directed mutagenesis (Stratagene, San Diego, CA, USA). The LDHA-wt or LDHA-mut vectors were co-transfected with either miR-200c mimics or scramble into T24 cells. The pMIR-REPORT β-galactosidase vector was transfected as a control. Luciferase activity was measured in cell lysates 48 hours after transfection using a Dual-light Luminescent Reporter Gene Assay Kit (Applied Biosystems, Foster City, CA, USA). The results were normalized to β-galactosidase activity.

### Western blotting

Western blotting was performed as described previously. The anti-LDHA and anti-β-actin antibodies were purchased from Santa Cruz Biotechnology (Santa Cruz, CA, USA).

### Xenograft mouse model

The protocol for the mouse xenograft study was reviewed and approved by the Institutional Animal Care and Use Committee of the Guangzhou Medical University. A total of 1 × 10^6^ T24 or 5637 cells were injected into the mice subcutaneously. The mice were randomly assigned to the scramble or miR-200c mimic groups (four mice per group) once tumors reached approximately 50 mm^3^. All mice received daily intratumoral injections for 3 weeks. After 28 days, the mice were sacrificed and necropsies performed. Tumors were weighed and miR-200c levels analyzed by qRT-PCR.

### Statistical analysis

Comparisons between groups were analyzed with t tests. Paired Student's t tests were used to compare mRNA or miRNA levels between bladder cancer and corresponding normal tissue. Two-tailed Pearson correlation tests were used to evaluate the correlation between miR-200c and LDHA expression. Data are expressed as the mean ± standard error of the mean (s.e.m). A *P* < 0.05 was considered statistically significant.
